# Identification of Pre-Heart Failure in Early Stages: The Role of Six Stages of Heart Failure

**DOI:** 10.3390/diagnostics14232618

**Published:** 2024-11-21

**Authors:** Monika Jankajova, Ram B. Singh, Krasimira Hristova, Galal Elkilany, Ghizal Fatima, Jaipaul Singh, Jan Fedacko

**Affiliations:** 1 First Department of Cardiology, East-Slovak Institute of Cardiovascular Diseases, 04011 Kosice, Slovakia; mjankajova@gmail.com; 2 Halberg Hospital and Research Institute, Moradabad 244001, India; rbs@tsimtsoum.net; 3 Department of Cardiology, Center of Cardiovascular Diseases, 1309 Sofia, Bulgaria; khristovabg@yahoo.com; 4 Gulf Medical College, Ajman 4184, United Arab Emirates; galal.elkilany@gmail.com; 5 Chronobiology Laboratory, Era University, Lucknow 226003, India; ghizalfatima8@gmail.com; 6 School of Pharmacy and Biomedical Sciences, University of Central Lancashire, Preston PR1 2HE, UK; jaipsingh10@gmail.com; 7 Department of Gediatrics, PJ Safaric University, 04001 Kosice, Slovakia

**Keywords:** myocardial dysfunction, hypertrophy, heart enlargement, stages of heart failure, speckle-tracking echocardiography

## Abstract

Despite increased availability of effective drug therapy for treatment of heart failure (HF), the morbidity and mortality in chronic heart failure (CHF) are unacceptably high. Therefore, there is an urgent need to ascertain new imaging techniques to identify early sub-clinical forms of cardiac dysfunctions, to guide early relevant treatment. It seems that all the behavioral risk factors—such as tobacco, alcoholism, Western-type diet, sedentary behavior and obesity, emotional disorders, and sleep disorder are associated with early cardiac dysfunction, which may be identified by speckle-tracking echocardiography (STE). Cardiac remodeling can also occur chronologically in association with biological risk factors of CHF, such as diabetes mellitus (DM), hypertension, cardiomyopathy, valvular heart disease, and coronary artery disease (CAD). In these conditions, twisting and untwisting of the heart, cardiac fibrosis, and hypertrophy can be identified early and accurately with 2-Dimentional (2D) and 3D echocardiography (2D echo and 3D echo) with tissue Doppler imaging (TDI), strain imaging via STE, and cardiac magnetic resonance imaging (CMR). Both 2D and 3D echo with STE are also useful in the identification of myocardial damage during chemotherapy and in the presence of risk factors. It is possible that global longitudinal systolic strain (GLS) obtained by STE may be an accurate marker for early identification of the severity of CAD in patients with non-ST segment elevation MI. Left ventricular ejection fraction (LVEF) is not the constant indicator of HF and it is normal in early cardiac dysfunction. In conclusion, this review suggests that GLS can be a useful early diagnostic marker of early or pre-cardiac dysfunction which may be treated by suitable drug therapy of HF along with the causes of HF and adhere to prevention strategies for recurrence. In addition, STE may be a superior clinical tool in the identification of cardiac dysfunction in its early stages compared to ejection fraction (EF) based on conventional echocardiography. Therefore, it is suggested that the chances of either stalling or reversing HF are far better for patients who are identified at an early stage of the disease.

## 1. Introduction

Chronic heart failure (CHF) continues to be associated with considerable worldwide morbidity and mortality [1]. Early diagnosis in the first 2–3 stages of the six stages of HF may be useful in the reversal of HF [2,3]. The Cleveland Clinic and other experts have classified HF into four stages—A, B, C, and D [3,4,5,6]—which are different from the New York Heart Association (NYHA)’s classes I–IV [1]. There are gaps in the knowledge about when to institute drug therapy despite the increased availability of four pillars of pharmaco-therapy for the treatment of HF [7,8]. Early identification of HF requires the establishment of new imaging techniques to guide treatment of patients presenting with biological risk factors—such as obesity, diabetes mellitus (DM), hypertension (HTN), and coronary artery diseases (CAD)—as well as those with acute coronary syndromes [9]. Apart from biological risk factors, behavioral risk factors may also cause pre-heart failure (PHF), which may be diagnosed via speckle-tracking echocardiography (STE) [9,10,11]. Advanced cardiac imaging has progressed significantly which serves as a major diagnostic, as well as a prognostic tool [11,12,13,14]. It seems that patients with CHF need continuous follow-up along with imaging such as 2D echo or STE and stress imaging to identify HF in early stages. Echocardiography can guide therapy and related procedures concerning intervention for the better prognosis of HF patients [11,12]. This communication was designed to highlight the important role of biomedical, biological, and behavioral risk factors in an early stage of HF or PHF and moreover to emphasize on the significance of such cardiac clinical tools and imaging techniques as STE, echocardiography, and CMR to aid early diagnosis process leading to early treatment of the disease.

## 2. Risk Factors of Pre-Heart Failure (PHF)

**Figure 1:**
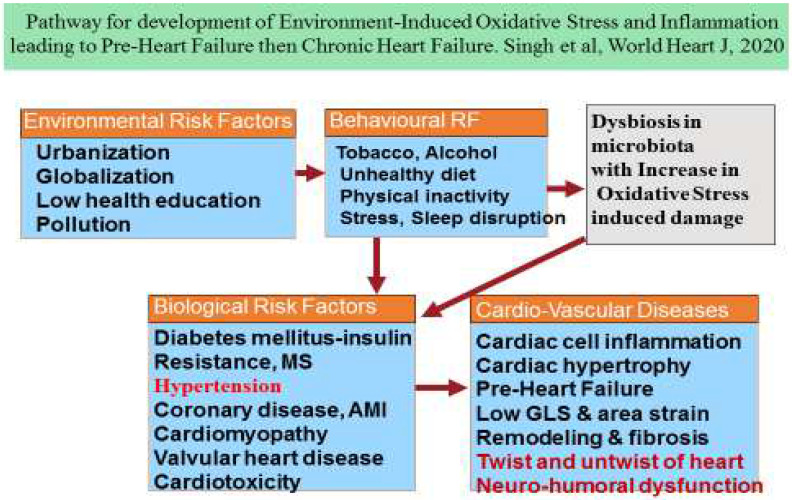
Pathways for the development of cardiac dysfunction leading to pre-heart failure [10].

There is much evidence that an increase in the dysfunction of myocardial tissue predisposes to reduced pumping activity, which worsens the clinical and biological functions of the heart, because cardiac muscle pumps less blood to organs, leading to the various stages of HF [3,4,5,6,7]. In the treatment of HF, the goal is to reduce worsening of failure from progression through the stages, or to slow down the progression of failure to advanced stages of HF [4,5,6,7]. It is crucial to target HF before its clinical manifestation, at the stage of PHF, during asymptomatic phase. There is an unmet need to identify PHF in the early stage in the population via 2D and 3D STE among subjects predisposed to risky health behaviors and risk factors of PHF. The risk factors of PHF could be behavioral as well as biological. However, the risk of these risk factors can be variable due to presence of protective factors, which may blunt the adverse effects of these risk factors on cardiac function. Figure 1 illustrates the pathways for the development of cardiac dysfunction leading to pre-heart failure, while Table 1 shows the risk factors and protective factors of HF.

## 3. Effects of Behavioral Risk Factors on Development of Pre-Heart Failure

Table 2 illustrates the effects of behavioral risk factors on pre-heart failure of the six stages of HF via speckle-tracking echocardiography. The six stages of HF have been proposed to identify earliest changes in cardiac dysfunction because early diagnosis may be useful in the primordial prevention [13,14]. There is much evidence that behavioral risk factors can independently predispose PHF [9,10]. It seems that STE, via 2D and 3D echo, has demonstrated abnormalities in global longitudinal strain (GLS) and area strain, indicating PHF in patients with primary risk factors [1,2,3,4]. There is an unmet need to conduct STE studies among patients with these risk factors for early diagnosis of PHF [9,10]. These patients with behavioral risk factors may not reveal any clinical manifestations of HF, and 2D echo may be within normal limits [9,10]. Early diagnosis at the stage of PHF, at first two stages of the six stages of HF, may be useful while treating for the reversal of HF [9,10].

Previous studies indicated that Western-type diets rich in sweetened products, tobacco and alcoholism, short sleep, stress, and depression are known to be associated with increased risk of cardiovascular diseases (CVDs), including HF [15,16,17,18,19,20,21,22,23,24,25,26,27]. All these risk factors are known to cause oxidative dysfunction of the myocardium leading to HF [10,18]. Using 2D-STE myocardial imaging tobacco smoking has been confirmed to have adverse effects on the myocardium [17,28,29]. The global longitudinal strain (GLS), as assessed by 2D speckle-tracking echocardiography, was also significantly reduced in smokers (*p* < 0.05) [28,29]. It is possible that chronic tobacco smoking alters long-axis diastolic and systolic functions in healthy hearts with influence on the right ventricle, which may be detected early with 2D STE [28,29]. In another study of 100 healthy males (50 smokers and 50 non-smokers), all subjects underwent echocardiography to compare diastolic function of the left (LV) and right ventricles (RV) and examined the chronic and acute effects of smoking [28]. The atrial late diastolic mitral inflow velocity (Am) and atrial late diastolic septal mitral annular velocity were high in smokers compared to those of the control group before smoking. After 5 min, isovolumetric relaxation time (IVRT) was prolonged, and septal mitral annular velocity showed an increase as a sign of left ventricular diastolic dysfunction. In the right ventricle, the changes that occurred were similar and in favor of acute diastolic dysfunction. After 30 min, early diastolic tricuspid inflow (Et) decreased and septal mitral annular velocity and late diastolic tricuspid inflow (At) remained high. After 5 min, diastolic blood pressure increased but returned to normal after 30 min. However, pulmonary arterial pressure did not change before and after smoking. Smoking for a longer time causes left ventricular diastolic dysfunction, whereas acute intake causes left and right ventricular diastolic dysfunction [28]. Similar results have been found due to adverse effects of acute smoking in an earlier study [29].

Emotional stress may be a risk factor for CVDs, including HF [19,20,21,22,23,24,25]. In a cohort study, among 82,695 men, during a follow-up of mean 7.8 years (646,989 person-years), 3473 males had HF [19]. After controlling for time in sedentary behavior, HTN, DM, dyslipidemia, tobacco smoking, body mass index (BMI), and diet, the hazard ratio (95% confidence interval (CI)) of HF in the category of lower physical activity compared to subjects in the highest category was 1.52 [19]. Those in the category of medium physical activity also had greater risk (HR, 1.17 (95% CI, 1.06–1.29)). Medium sedentary time also conveyed risk (hazard ratio, 1.13) [19]. Sedentary behavior may be a risk factor for CHF. Among patients either with or without CAD, greater mental distress was associated with higher cardiovascular events in the future [22,23,24,25].

Increased heart rate can interfere with normal heart function and increase the risk of sudden cardiac arrest in patients with HF. Mental stress may also increase blood pressure in HTN and can lead to coronary disease, as well as weakening of the cardiac muscle and the immune system, leading to HF. There is much evidence that depression may predispose to increased platelet reactivity, decreased heart variability, and increased pro-inflammatory cytokines, which are all risk factors for HF [22,23,24]. In a cohort study of 136,637 patients with mental disorders—along with patients of post-traumatic stress disorder (PTSD), acute stress reaction, adjustment disorder, and other stress reactions—in which all subjects were followed up for 27 years and including 171,314 unaffected full siblings of these patients and 1,366,370 matched unexposed people from the general population [23]. The crude incidence rates of any CVDs were 10.5, 8.4, and 6.9 per 1000 person years during the follow-up of 27 years. In sibling-based comparisons, the HR for any CVDs was 1.64, with the greater subtype specific HR observed for HF (6.95, 1.88 to 25.68), during the first year after the diagnosis of any stress related disorder [23]. Beyond one year, the HRs became lower (overall 1.29), ranging from 1.12 for arrhythmia to 2.02 for artery thrombosis/embolus. It is possible that stress disorders had strong association with all types of CVDs, including HF, independent of family history of somatic/psychiatric diseases, and psychiatric comorbidity.

In a case–control study, the borderline personality disorder (BPD) group (*n* = 50) had a higher prevalence of cardiovascular risk factors for CVD compared to the control group (*n* = 50) [25]. Subjects in the BPD group revealed associations with chronic stress, major risk factors, and abnormalities in the myocardial wall motion as revealed by STE. It seems that chronic stress influences the structures in the cortico-limbic system and the nuclei responsible for the regulation of autonomic function, eliciting an imbalance of sympathetic and vagal activity, leading to adrenergic activation and withdrawal of vagal activity [24]. The adrenergic terminals of the nerves are connected to cardiac cells in a form of quasi-synasis, resembling the neuro-cardiac junction. The release of norepinephrine is increased, during chronic stress, leading to increased stimulation of cardiomyocytes via β_1_-adrenergic receptors, causing mainly calcium dynamics and β_2_-adrenergic receptors, which control housekeeping functions. There is impairment of the circadian rhythm of cardiomyocytes with elongation of the catabolic compared to the anabolic nocturnal phase, leading to a depletion of cell energy storage, with a reduced turnover of cell constituents [24]. The coupling concerning cardiomyocytes declines while there is an increase in the coupling between cardiomyocytes and fibroblasts, with alteration in the shape and velocity of action potential, fibroblast activation, and deposition of extracellular matrix. These alterations can predispose stress-related arrhythmias and cardiomyopathy.

There is profound adverse impact of disturbed sleep, such as in shift work, on circadian rhythms and of sleep on the cardiovascular system [26,27]. In a clinical study involving 784 consecutive patients, 165 patients had central sleep apnea, 139 had no sleep-disordered breathing; the remainder had obstructive sleep apnea (OSA) [26]. The rate ratio of cardiac readmissions in 6 months was 1.53; *p* = 0.03) in patients with central sleep apnea compared to no sleep-disordered breathing. The results revealed that severe OSA was also an independent predictor of readmissions with an adjusted rate ratio of 1.49 (*p* = 0.04). The impact of breathing due to sleep disorder on cardiac readmissions in HF showed that central sleep apnea was an independent risk factor for 6-month cardiac re-hospitalization. The effect of central sleep apnea on admissions for CVDs including HF exceeded that of all known predictors of readmissions [27].

## 4. Mechanisms and Pathophysiology of Pre-Heart Failure (PHF)

The mechanisms of adverse effects may be due to micro-inflammation in the mitochondrial signaling pathway [30], redox signaling in the cardiac myocyte [31] and Western diet induced cardiac ceramide [32]. These adverse effects may be inhibited by antioxidants either present in the diet or by antioxidant administration [33]. Oxidative dysfunction and inflammation in the cardiac tissues predisposes intracellular Ca^2+^ overload, via activation of proteases and phospholipases, mitochondrial dysfunction, and. alterations in cardiac gene expression and accompanied with alterations in the molecular structure, biochemical composition and physiology of various subcellular organelles. In turn, all these lead to pathological subcellular remodeling or myocardial deformation.

## 5. Effect of Biological Risk Factor on Pre-Heart Failure

Acute coronary syndromes (ACS); in-particular myocardial infarction (AMI), is the most common biological risk factor of PHF that may lead to CHF [34]. Other risk factors are obesity [11,35,36,37], cardiomyopathy [38], valvular heart disease [39], HTN [40,41,42], and DM [43,44,45]. On the other hand, AMI is a highly inflammatory state characterized with marked increase in catecholamine, cortisol and aldosterone, insulin, lipoprotein and brain natriuretic peptide. In turn, these induce acute reactants to fight the oxidative stress, which is damaging to cardiac cells, neurons, beta cells, and nephrons [34,35,46,47]. In a clinical study of a cohort of AMI survivors with ventricular dysfunction but no information of HF, plasma aldosterone levels estimated in the initial couple of days after confirmation was an independent indicator of remodeling [34]. Even in the absence of HF, higher plasma aldosterone concentrations—after ST-elevation myocardial infarction (STEMI) and non-STEMI—are associated with greater mortality. There is a consistent relationship between higher aldosterone concentrations and greater adverse remodeling. Remodeling may be due to the activation of both the cortisol and aldosterone limbs of corticosteroid synthesis, which may be responsible for myocardial fibrosis, leading to PHF [34].

## 6. Speckle-Tracking Echocardiography via 2D and 3D Echocardiography

It seems that 2D echocardiography is the commonest modality for imaging in the identification of cardiac hypertrophy [7,12,13,14]. However, in sub-clinical states, it is not able to detect subtle changes in cardiac structure. Recently, strain echocardiography, which is a new modality of echocardiography, has been found to identify subclinical dysfunction of the myocardium through measurement of intrinsic deformation of the myocardium. It is known that the co-existing abnormal conventional echocardiographic findings, such as LV systolic and diastolic dysfunction, abnormal LV geometry and LVH, and dilatation of the left atrium, can cause poor prognosis associated with biological risk factors of PHF. Since STE is a non-invasive modality examined using 2D and 3D echocardiography, it can provide information about myocardial deformation. STE may be more accurate in the detection of early cardiac ischemia and dysfunction of the myocardium undetected by the conventional echocardiographic examination before onset of symptoms [7,12,13,14].

**Figure 2:**
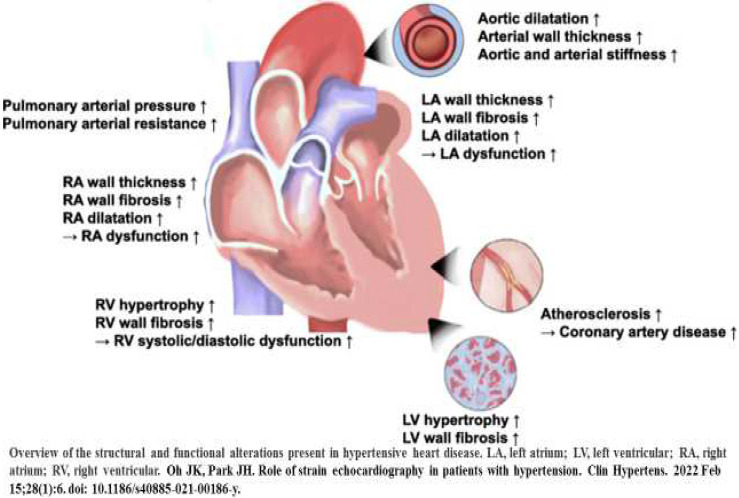
Diagram showing structural and functional alterations in various components of the heart due to risk factors of heart failure. (Adapted from Reference [40], Oh and Park, 2022).

The intrinsic myocardial performance can be measured through assessment of the strain rate and myocardial strain [40]. In addition, 2D-STE can be used to measure myocardial strain, by tracking ultrasonic speckles that are small footprints in the myocardium in 2-D images [48]. The myocardial speckles are tracked in each cardiac cycle, frame-by-frame, and the strain is calculated automatically by measuring the distances in the speckles [48]. It seems that deformation of the myocardium can show directions which include radial strain, longitudinal strain (LS), and circumferential strain (CS), where LS is commonly detected component of deformation assessed from averaging values. The left ventricular LS (LVLS) is under the influence of several factors, such as the geometry of chambers, the characteristics of myocardial tissue hemodynamic factors, and myocardial contraction synchrony. There may be a decline in LVLS due to tachycardia and increase in LV afterload, and the alteration in preload may be related to the change of LVLS [40]. Any increase in the thickness of LV walls or in the dilatation of the cavity of the LV may cause a decline in LS. There may be decline in the regional LS in the areas of infiltration as well as in the myocardial fibrosis after MI [40,48]. In the presence of a left bundle branch block, non-homogeneous myocardial activation can cause differences in the sequence of myocardial contraction of the ventricular septum and the LV lateral wall. In addition, age and gender can also affect LS. It is known that females have better LVLS values compared to males and that older adults have lower LS values than younger adults. Figure 2 shows the structural and functional alterations in various components of the heart due to risk factors of HF.

## 7. Effects of Hypertension (HTN) on Development of Pre-Heart Failure (PHF)

The early identification of cardiac changes due to HTN may allow reversal of the PHF [40,41,42]. HTN could be associated with complex and diverse alterations in the structure and function of the heart due to direct pressure on the cardiac cells. LV longitudinal strain during systole was impaired in patients with HTN and also in those with no LVH [41]. In LVH, radial strain may be decreased, and circumferential strain and twist may be increased. In hypertensive patients with normal EF, LS was significantly reduced, and basal-to-apical torsion became higher [42]. Higher serum concentrations of the tissue inhibitor matrix metalloproteinase-1, which is known to control myocardial collagen turnover, may be found among patients with impairment in the LS and increased torsion in the LV in patients with hypertension and normal LVEF. Excessive collection of collagens in fibroblasts may progress to myocardial fibrosis, leading to early LV contractile dysfunction. Since high blood pressure can impact all myocardial layers—including epicardial, mid-cardiac, and endocardial—LS may be lower than normal. It seems that epicardial LS could be an important predictor for CV events, including mortality and re-hospitalization in patients with hypertension.

## 8. Effects of Obesity, Diabetes, and Metabolic Syndrome on Pre-Heart Failure

In individuals with excess body weight, several pathophysiological processes can directly predispose the risk of PHF. These mechanisms are neuro-hormonal activation, hemodynamic alterations, hormonal effects of dysfunctional adipose tissue, ectopic fat deposition with resulting lip toxicity, and microvascular dysfunction. Overweight and obesity may indirectly cause PHF via causal increases in high BP, dyslipidemia, metabolic syndrome, and—most importantly—type 2 diabetes mellitus (T2DM) via producing insulin resistance [11]. Lower physical activity and fitness can further predispose risk of PHF in relation to obesity and insulin resistance (IR). These pathophysiological mechanisms may lead to myocardial-injury-induced cardiac remodeling, which is first physiological but later becomes pathological. The pathological remodeling is reflected via abnormal cardiac biomarkers and cardiovascular (CV) function on STE myocardial imaging.

Recently, Galal and coworkers have reported the prediction of preclinical dysfunction of the myocardium among patients with obesity and diabetes with preserved ejection fraction, by use of STE and tissue Doppler imaging [11]. In their study of 240 subjects with obesity, the results indicate that patients with obesity with T2DM should be advised to undertake early tissue Doppler imaging and STE for early diagnosis of decreased cardiac diastolic and systolic dysfunctions and cardiomyopathy [11]. These conditions are likely to be missed by conventional echocardiography. Significant differences in regional and global strains were also identified between the severely obese diabetic (BMI ≥ 35) (global longitudinal pre-systolic strain, −13) patients compared to less obese subjects.

The CARDIOBESE study included 100 obesity patients (body mass index (BMI) ≥ 35 kg/m^2^) without known CVD and 50 age-matched and gender-matched non-obese controls (BMI ≤ 30 kg/m^2^) [36]; 59 showed subclinical cardiac dysfunction. The data reveal that 57 patients had decreased global GLS and that two patients with normal GLS had either diastolic dysfunction or increased brain natriuretic peptide (BNP). In patients with obesity, autonomic dysfunction was observed as a significant independent risk factor for preclinical heart dysfunction. There was a greater prevalence (61%) of cardiac dysfunction in obesity patients—which was subclinical and without known CVD—diagnosed by GLS. In another case study, including 589 patients with obesity and 100 non-obese controls [37], the value of GLS was significantly lower in obesity compared to non-obese controls (*p* < 0.001). The values of global work index, efficiency, constrictive work, and wasted work in obese patients were also significantly lower compared to non-obese controls (*p* < 0.05), while global wasted work was significantly higher than that in nonobese controls (*p* < 0.001). It seems that higher BMI in patients with obesity may produce a greater risk of subclinical LV systolic dysfunction with coexisting normal LVEF [37].

The STE may also be helpful in the identification of subclinical signs of cardiomyopathy due to diabetes, although significant heterogeneity in the strain values has been reported in the literature [43]. A meta-analysis involving 41 valid studies, which included 6668 subjects with T2DM and 7218 controls, was carried out. The mean and mean difference (MD) in each group were pooled for left ventricular (LV) GLS, LV global circumferential strain (GCS), LV global radial strain (GRS), LV longitudinal systolic strain rate, left atrial reservoir strain (RS), and right ventricular GLS for the assessment. Interestingly, those patients with T2DM had 2 units lower LVGLS overall compared to control subjects (17.5%, vs. 19.5, mean difference = −1.96). The values of other strains were also lesser in patients with T2DM: LVGCS (mean difference = −0.89; LVGRS (mean difference = −5.03; LVSR (mean difference = −0.06; LARS (mean difference = −8.41; and right ventricular GLS (mean difference = −2.41. Higher body mass index was identified as the single greatest contributor to worse LVGLS, LVGCS, and LV strain rate. There was a worsening of right ventricular GLS in patients with higher HBA1c. Myocardial strains were decreased in the whole heart in T2DM patients. The largest decline was found in LA reservoir strain, followed by right ventricular GLS and LVGLS. In patients with T2DM, a greater body mass index was also associated with worse LV strain values. Table 3 illustrates the six stages of chronic heart failure among patients with biological risk factors indicating pre-heart failure via speckle-tracking echocardiography.

In a case control study, 150 subjects with mean age; 62 ± 10 years, 82 males (55%) with metabolic syndrome were included, and they were divided into two groups based on the presence of T2DM [44]. Apart from liver fibrosis, LV diastolic dysfunction was found in 52% of the patients with diabetes and in 36% of non-diabetic patients with metabolic syndrome (*p* = 0.04). In subjects with diabetes, there was an increased left atrial stiffness (40% vs. 24%, *p* = 0.03) and decreased left ventricular GLS (47% vs. 16%, *p* < 0.0001) in nondiabetics via 2D-STE. The link between stiffness of the liver and subclinical cardiac dysfunction detected by 2D-STE in metabolic syndrome patients with T2DM is justified. These parameters derived from left atrial and left ventricular 2D-STE may have greater sensitivity compared to the older measurements and a substantial link to fibrosis of the liver.

In a case study, four groups of subjects were designated as Group I (*n* = 56), with T2DM and moderate HTN [45]; Group II (*n* = 52), with T2DM with normal blood pressure; Group III (*n* = 54), with grade II HTN without diabetes; and Group IV (*n* = 30, control), healthy subjects. The results showed that diastolic dysfunction was significantly (*p* < 0.05) more common in patients with a combination of T2DM and moderate HTN in contrast to patients with diabetes without HTN or those with HTN without diabetes. The combination of T2DM and HTN was associated with an increase in the longitudinal global deformation of the left ventricle compared with patients who had only one of these diseases (*p* < 0.05). A decrease in the global area strain, an early marker of LV systolic dysfunction, was expressed (*p* < 0.05) in patients with T2DM, regardless of the presence of concomitant HTN [45]. This study further shows the utility and necessity of tissue Doppler echocardiography and STE in the diagnosis of subclinical HF. The results reemphasize that high prevalence of subclinical systolic–diastolic LV dysfunction in T2DM may be aggravated in the presence of concomitant HTN in patients without obvious clinical signs of PHF and other CVDs. Although twist dysfunction is the beginning of PHF [48], there is a need to examine GLS in these patients [49].

## 9. Oxidative Function and Dysfunction in the Pathogenesis of Pre-Heart Failure

Risk factors may cause inflammation due to oxidative dysfunction in the cardiac ultra-structure. However, it is not well known how oxidative function of the myocardium and oxidative dysfunction can cause physio-pathological remodeling, predisposing to HF [2,48,50]. During metabolism, a small amount of reactive oxygen species (ROS) is generated in the cells for cell signaling, which may be spontaneously modulated by the endogenous antioxidant defenses to perform the oxidative function of the myocardium. Protective factors such as Indo-Mediterranean diets and physical training without any harmful health behaviors may be helpful in sustaining the oxidative functions of the heart and delay the development of cardiac cell dysfunction responsible for PHF. Oxidative stress is characterized by an imbalance in the generation of ROS and the availability of endogenous antioxidant enzymes and other defenses [18]. In the myocardial cells, the development and progression of maladaptive myocardial remodeling could be an early stage of HF with decline in GLS. Cardiac electrophysiology could be impaired by targeting contractile machinery and cardiac components via the dysfunction of proteins that are crucial to excitation–contraction coupling (ECC), including sodium channels, L-type calcium channels, potassium channels, ryanodine receptor (RyR), sarcoendoplasmic reticulum calcium ATPase (SERCA pump), and the sodium–calcium exchanges [2,18,51,52].

**Figure 3:**
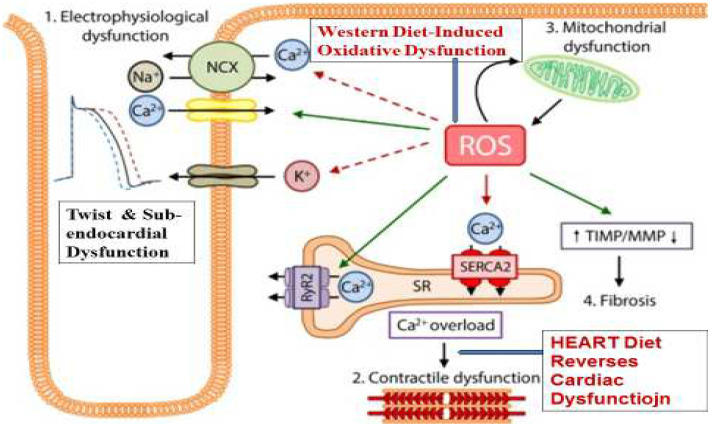
Oxidative dysfunction in the heart due to Western diets and other risk factors leading to decreases in antioxidant defenses causes mitochondrial dysfunction, leading to electrophysiological dysfunction with twist and sub-endocardial dysfunction. Exogenous antioxidants (HEART diet) and lifestyle modification may improve antioxidative function with reduced Ca overloading and reversal of mitochondrial and electrophysiological dysfunction. (Modified from Reference [18], Vander Pol A, Euro J Heart Failure 2019, under the license http://creativecommons.org/licenses/by-nc/4.0/, accessed on 1 January 2024).

The biological alterations may begin with physiological cardiac cell remodeling to pathological remodeling due to uncontrolled increase in oxidative stress, inflammation and infiltration of fibrosis, which may increase many folds due to presence of other behavioral and biological risk factors [9,10,11]. In the presence of the risk factors, the generation of ROS could be enormous compared to the buffering ability of the cellular endogenous antioxidant defenses [2,7,48,50]. The increased production of ceramide due to high sugar and high fat in the diet also induces high advanced glycation end-products (AGE) and triamino-methyl-N-oxide (TMAO), which can predispose oxidative dysfunction due to over-loading of cytosolic calcium in the cardiac cells. These biological alterations may result in damage to protein and DNA, peroxidation of lipids with an increase in troponin T [2], and cell damage due to apoptosis [2,51,52]. Figure 3 reveals oxidative dysfunction in the heart due to Western diets and other risk factors with a decrease in antioxidant defenses, causing mitochondrial dysfunction and leading to electrophysiological dysfunction due to derangement in cation-transporting proteins (including L-type calcium channels, potassium channels, RyR). SERCA and NCX also showed twist and sub-endocardial dysfunction.

Oxidative dysfunction may alter the activity of RyR and SERCA, which are associated with cellular calcium mobilization. In turn, this can lead to a decline in myofilament calcium sensitivity and protein function as well as in energy metabolism, resulting in diastolic dysfunction [51]. Thus, oxidative dysfunction induces loss of cardiac cells due to apoptosis, development of fibrosis, and alteration of physiological remodeling to pathological remodeling of the myocardium. Oxidative dysfunction can predispose a pro-fibrotic function as a physiological adaptation by causing the proliferation of cardiac cells, such as fibroblasts and matrix metallo-proteinases, for extracellular remodeling, initiating the hypertrophy of the heart [2,51,52]. The hypertrophy of the heart may be in multiple stages, depending upon the extent of oxidative dysfunction in the cardiac cells. These stages of PHF may be classified into six stages of HF, depending upon the effects of pathological remodeling on cardiovascular hemodynamics and electrophysiological dysfunction causing dysregulation in the twist and sub-endocardial function. Table 4 shows the clinical and echocardiographic features of six stages of heart failure proposed in patients with risk factors of heart disease.

It is crucial to know the biology of the six stages of HF via an understanding of twist function which may be the earliest change during oxidative dysfunction of the heart and that may require grading of the LV twist [39,53,54]. It is clear from the apex of the left ventricle that the cardiac twist or torsion indicates the mean longitudinal gradient of the net difference in the clockwise and counterclockwise rotation of the apex and base of the LV [55,56,57]. There is a storage of the cell energy during deformation of the LV twist in the sub-endocardial fiber matrix to complete the recoil. It is possible that it may facilitate release of restoring forces, causing further deformation in the recoil of twist and thus contributing to diastolic relaxation of the LV with early diastolic filling [48,55,56,57]. Interestingly, despite the normal ejection fraction, the systolic function could also be dysfunctional. Therefore, there may be greater subtle defects with a decline in the left ventricular systolic long axis at earlier stages of PHF. Bath et al. [12] have also emphasized the above points about the earlier diagnosis before the clinical manifestations of CHF. Lastly, HF may be reversed if behavioral risk factors—in particular, nutritional factors—are duly considered during the management of HF [58,59,60,61]. The clinical value of GLS is being gradually established to ascertain the atrial function [62] in patients with acute myocardial infarction [63,64].

## 10. Modalities in Assessing Heart Failure

It seems that conventional echocardiography (CE) with color-encoded Doppler, pulsed, and continuous-wave Doppler are the best modalities for non-invasive imaging in the quantitative assessment of valvular heart diseases and cardiomyopathy [12,14]. However, left ventricular GLS is now considered as a more sensitive measure of LV myocardial systolic function compared to LVEF. Therefore, recent guidelines for diagnosis and treatment of valvular heart disease, CAD and cardiomyopathies emphasize the value of LVEF as one of the main parameters for the clinical decision-making [1,14]. Despite these considerations, it has been found that left ventricular GLS is a classical sensitive marker of LV contractility and inotropic state [1,2,3]. Therefore, GLS could be a better indicator of prognosis compared to LVEF in patients at increased risk of HF. Recent techniques for imaging, such as strain and computerized magnetic resonance, can quantify structural alterations—including cardiac mass, fibrosis, shape, and functional parameters; EF; blood flow; and other alterations most accurately with improved temporality. It is possible that the same information can have tremendous benefit in the planning of treatment and monitoring of health care in these patients. STE via 2D echo and 3D echo, and computerized magnetic resonance imaging (CMSI) may be beneficial in the identification of ACC Stage A, B, and C patients by detecting abnormal subclinical myocardial dysfunction and deformation at the earliest stages of PHF.

## 11. Limitations and Disadvantages

STE via the 3D echo is a novel technique, but it has limitations due to lower temporal resolution compared with 2D echo. Apart from this, the acquisition requires excellent windows and quality of image. The normal metrics in STE are quite vendor-sensitive; therefore, the normal values range for GLS, range from −18.5% to −22.0%. Moreover, there are gender-specific differences in the normal values of GLS, global circumferential strain, and central circumferential strain. Another disadvantage is that speckle motion is measured more at the endocardial border and less in the myocardium. In addition, the cardiologist/physician must understand and standardize the methodology and generate data for normal values that can be standardized for clinical use for each modality and across modalities to factor these into guidelines.

## 12. Prevention Strategies for Pre-Heart Failure and Consequences for Clinical Cardiologists

The pillars in the prevention strategies for pre-heart failure are related to lifestyle changes, the regulation of blood glucose levels, modification of risk factors for the development of CVDs and the treatment of HF combined with an element of psychological intervention to adhere to daily treatment and follow up educational programs by health professionals. Achieving euglycemia reduces the risk of major cardiovascular events, such as myocardial infarction or stroke, and the likelihood of developing pre-heart failure [65]. Other strategies involve early healthy screening tests, smoking cessation, limiting alcohol consumption and salt intake, reducing body weight, and avoiding obesity by eating moderate amounts daily and regular aerobic exercise are the cornerstones in terms of lifestyle change. The susceptible patient must also focus on a nutritious diet, such as the Mediterranean diet, which is low in sodium, fats, and carbohydrates but high in fruits, fibers, vegetables, and whole grains—foods which are rich in antioxidants—to manage blood pressure and weight [66]. Regular physical activity, as recommended by the Physical Activity Guidelines for Americans, plays a vital role, with a suggestion of at least 150 min of moderate aerobic exercise weekly to maintain cardiovascular health [67]. Achieving and maintaining a healthy weight range, ensuring adequate sleep, and reducing or managing stress levels and blood pressure are crucial. It is also important to take medication as prescribed and to avoid medications which may trigger arrhythmia.

Missed opportunities of early diagnosis are incidents in which different actions by the clinical cardiologist involved could have resulted in more desirable events. Thus, early screening and diagnosis can give the cardiologists more time to find a treatment that can keep the symptoms of the patients in check and prevent total HF and other health issues. The cardiologist must undertake a blood test for early markers, an electrocardiogram (ECG) to determine ant derangement in electrical activity of the heart and follow up with an echocardiogram or ultrasound technique to determine any dysfunction of the sound wave and valves of the myocardiogram. It is well established that HF is a complex clinical syndrome presenting as symptoms and signs common to other diagnoses, frequently in patients with multiple co-morbidities. HH itself is not a diagnosis, but it is the common clinical presentation of a variety of cardiac conditions. Correct diagnosis involves a combination of the clinical presentation, the results from general and specific investigations and diagnoses, and the ability of the clinical cardiologist to analyze the information for early intervention and treatment. Misdiagnosis can occur at any level during the development of HF and can occur because of factors related to the patient, the cardiologist and the health systems. As such, delayed diagnosis can lead to early—as well as excess—morbidity and mortality in these patients [40].

## 13. Conclusions

The continuous progression of the disease, occurring in PHF, begins with cardiac and neuro-humoral dysfunction initiated by behavioral and biological risk factors of HF. It seems that 2D echocardiography is considered the primary non-invasive imaging tool for the diagnosis of patients with CHF and for the assessment of prognosis. Both 2D echo and 3D echo have been used as the cornerstone or gold standard in the assessment of cardiomyopathy and valvular heart disease (VHD) and the method of choice in identifying and evaluating the morphology and severity of aortic, pulmonary, tricuspid, and mitral valve diseases. The combined use of 2D echo and 3D echo and STE has improved image quality and early diagnosis of cardiac dysfunction by identifying GLS and area strains as well as twist and untwist of the heart. Since ejection fraction does not indicate HF in many patients with PHF, and it is inconclusive in cardiac-dysfunction-causing PHF, there is an urgent need to develop and create evidence on the role of cardiac strain in the early diagnosis of HF. It is possible that GLS by STE analysis could be an accurate method in the early diagnosis of the severity of CAD in patients presenting with non-ST segment elevation (NSTSE) myocardial infarction as well as in subjects with behavioral risk factors and biological risk factors, who are likely to have PHF.

## Figures and Tables

**Table 1:** Risk factors and protective factors of heart failure.

Behavioral Risk Factors	Biological Risk Factors	Protective Factors
Western diet	Hypertension	Mediterranean-style diet
Tobacco use	Diabetes mellitus	Exercise training
Alcoholism	Coronary artery disease	Yoga posture
Mental disorders	Metabolic syndrome	Circadian restricted, day eating
Cardiotoxic drugs; cancer drugs	Rheumatic heart disease	Intermittent fasting
Sleep disorders	Cardiomyopathy	Optimal sleep
Sedentary behavior	Obesity	Meditation and prayer

**Table 2:** Effects of behavioral risk factors on pre-heart failure of the six stages of heart failure via speckle-tracking echocardiography.

Stages of HF	Tobacco Smoking	Alcoholism	Western Diets	Sleep Disorders	Emotional Disorders	Sedentary Behaviors
**Stage A**	Normal GLS	Normal GLS	Normal GLS	Normal GLS	Normal GLS	Normal GLS
**Stage B**	Normal GLS	Preserved GLS	Normal GLS	Preserved GLS	Normal GLS	Normal GLS
**Stage C**	Preserved GLS	Preserved or reduced GLS	Preserved GLS	Preserved or reduced GLS	Preserved GLS	Preserved GLS
**Stage D**	Slightly Reduced GLS	Reduced GLS	Slightly Reduced GLS	Reduced GLS	Slightly Reduced GLS	Slightly Reduced GLS
**Stage E**	Moderately reduced GLS	Heavily reduced GLS	Moderately reduced GLS	Heavily reduced GLS	Heavily reduced GLS	Heavily reduced GLS
**Stage F**	Heavily reduced GLS	Heavily reduced GLS	Heavily reduced GLS	Heavily reduced GLS	Heavily reduced GLS	Heavily reduced GLS

GLS = global longitudinal strain, HF = heart failure.

**Table 3:** Six stages of chronic heart failure among patients with biological risk factors indicating Pre-heart failure via speckle-tracking echocardiography.

Stages of Chronic Heart Failure	Obesity	Diabetes	Hypertension	CAD
**Pre-heart Failure Stage 1** Dysfunction of the twist	Normal or slightly increased LVT, UNTR	Normal or slightly decreased GLS	Increased LVT	Slightly decreased LVT, UNTR
**Pre-heart failure Stage 2** Sub-endocardial dysfunction	Slightly decreased GLS, LVT, UNTR	Slightly decreased GLS, LVT, UNTR	Increased LVT, delayed UNTR Decreased GLS	Decreased LVT, GLS
**Pre-heart failure Stage 3** Asymptomatic physio-pathological remodeling+	Slightly increased LVT, preserved GLS	Moderate decreased GLS, LVT, UNTR	slightly increased LVT, delayed UNTR, preserved GLS	Decreased LVT, GLS
**Complete heart failure Stage 4**, LVH, symptomatic, diastolic dysfunction	Decreased LVT, UNTR, depressed GLS	Decreased GLS, LVT, UNTR	Preserved LVT, delayed or decreased UNTR, decreased GLS	Decreased LVT, decreased and delayed UNTR, decreased GLS
**Complete heart failure Stage 5**, Severe LVH	Decreased LVT, UNTR, GLS	Decreased LVT, UNTR, GLS	Decreased LVT UNTR, GLS	Decreased LVT, UNTR, GLS
**Complete heart failure Stage 6**, Severe LVH	Decreased LVT, UNTR, GLS	Decreased LVT, UNTRGLS	Decreased LVT, UNTR, GLS	Decreased LVT, UNTR, GLS

LVT = left ventricular twist, UNTR = untwist rate, CAD = coronary artery disease, GLS = global longitudinal strain, LVH = left ventricular hypertrophy.

**Table 4:** Clinical and echocardiographic features of six stages of heart failure proposed in patients with risk factors of heart disease.

Stages (HT on HF)	Manifestations	2D Echocardiography	2D Speckle-Tracking Echo	3D Speckle-Tracking Echo
Stage A	Mild to moderate Oxidative dysfunction, neuro-humoral dysfunction begins.	Increasing filling pressure with abnormal relaxation.	Dysfunctional untwist rate. LA strain reduced.	Dysfunctional untwist rate, LA strain reduced.
Stage B	Moderate oxidative dysfunction, hyper-rotation. Sub-endocardial dysfunction.	Dysfunction of systole.	Dysfunctional untwist rate and increased diastolic pressure. LA strain decreased.	Dysfunctional untwist rate and increased diastolic pressure. LA strain decreased.
Stage C, PHF	Asymptomatic Physio-Pathological remodeling+.	EF% normal > 53%	Normal GLS −20–−23% ≥−27.0% area strain	Normal GLS −17–21% Normal AS −31–−36%
Stage D, PHF and HFpEF	Pathological remodeling disease without symptoms of HF but elevated Natriuretic peptide, dyspnea on exertion.	EF% ≥ 50% Systolic LV dysfunction.	EF% 40–49% Impaired GLS −16–20% Impaired GCS, GRS Impaired early diastolic SR, right ventricular LS, and global RV longitudinal SR.	Impaired GLS−16–20% Impaired AS −27–31%
Stage E, HFmrEF	Structural heart disease with symptoms of HF.	EF% 40–49% Grade 1, diastolic dysfunction.	Reduced GLS−12–16%, reduced GCS, GRS, treat with ACE, ARB.ARNI.	GLS ≤ −16% AS ≤ −27%
Stage F, HFrEF	Refractory class III HF.	EF% < 40%	All above GLS < −12%, treated with ARNI	GLS < −13% AS < −27%

HT = hypertension, HF = heart failure, AS = aortic stenosis, HFpEF = Heart Failure with Preserved Ejection Fraction, GLS = global longitudinal strain, GCS = global circumferential strain, GRS = global radial strain, LV = left ventricle, SR = strain rate, ARNI = angiotensin receptor neprilysin inhibitor, mrEF = mild reduction in ejection fraction (EF), r = reduction in EF, LA = left atrial (Adapted from Reference [2]).

## Data Availability

No new data were created or analyzed in this study. Data sharing is not applicable to this article.

## References

[1] Heidenreich P.A., Bozkurt B., Aguilar D., Allen L.A., Byun J.J., Colvin M.M., Deswal A., Drazner M.H., Dunlay S.M., Evers L.R. (2022). 2022 AHA/ACC/HFSA Guideline for the Management of Heart Failure: A Report of the American College of Cardiology/American Heart Association Joint Committee on Clinical Practice Guidelines. J. Am. Coll. Cardiol..

[2] Singh R.B., Fedacko J., Pella D., Fatima G., Elkilany G., Moshiri M., Hristova K., Jakabcin P., Vaňova N. (2022). High Exogenous Antioxidant, Restorative Treatment (Heart) for Prevention of the Six Stages of Heart Failure: The Heart Diet. Antioxidants.

[3] Stages of Heart Failure. https://my.clevelandclinic.org/health/diseases/17069-heart-failure-understanding-heart-failure#management-and-treatment.

[4] Muraru D., Niero A., Rodriguez-Zanella H., Cherata D., Badano L. (2018). Three-dimensional speckle-tracking echocardiography: Benefits and limitations of integrating myocardial mechanics with three dimensional imaging. Cardiovasc. Diagn. Ther..

[5] Congestive Heart Failure: Symptoms, Causes, Treatment, Types, Stages. https://www.webmd.com/heart-disease/guide-heart-failure.

[6] Mann D.L., Bristow M.R. (2005). Mechanisms and models in heart failure: The biomechanical model and beyond. Circulation.

[7] Halabi A., Yang H., Wright L., Potter E., Huynh Q., Negishi K., Marwick T.H. (2021). Evolution of myocardial dysfunction in asymptomatic patients at risk of heart failure. JACC Cardiovasc. Imaging.

[8] Rahamim E., Nachman D., Yagel O., Yarkoni M., Elbaz-Greener G., Amir O., Asleh R. (2021). Contemporary Pillars of heart failure with reduced ejection fraction medical therapy. J. Clin. Med..

[9] Singh R.B., Elkilany G., Fedacko J., Hristova K. (2024). The Six Stages of Chronic Heart Failure. Chronic Heart Failure.

[10] Singh R.B., Komatsu T., Lee M.C., Dewi M., Watanabe S. (2020). Effects of behavioral risk factors, with reference to smoking on cardiomyocyte dysfunction. World Heart J..

[11] Elkilany G.N., Merril E., Ajash H., Singh R.B., Elkilany G.Y., Allah S.B., Nanda N.C., Singh J., Kabbash I., Sozzi F. (2020). Prediction of preclinical myocardial dysfunction among obese diabetics with preserved ejection fraction using tissue doppler imaging and speckle tracking echocardiography. World Heart J..

[12] Baath A.S., Singh J., Elsaady A., Singh R.B., Elmahal M., Lohana P., Elkilany G.E. (2021). Advances in cardiac imaging via speckle tracking echocardiography and allied techniques: Early diagnosis of heart failure. World Heart J..

[13] Singh R.B., Fedacko J., Elkilany G., Hristova K., Palmiero P., Pella D., Cornelissen G., Isaza A., Pella D. (2020). 2020 Guidelines on Pre-Heart Failure in the Light of 2D and 3D Speckle Tracking Echocardiography. A Scientific Statement of the International College of Cardiology. World Heart J..

[14] Singh R.B., Sozzi F.B., Fedacko J., Hristova K., Fatima G., Pella D., Cornelissen G., Isaza A., Pella D., Singh J. (2022). Pre-heart failure at 2D- and 3D-speckle tracking echocardiography: A comprehensive review. Echocardiography.

[15] Singhal S., Singh R.B., Haruichi R., Takahashi T. (2017). Can high sugar diets induce chronic heart failure?. World Heart J..

[16] Tikellis C., Thomas M.C., Harcourt B.E., Coughlan M.T., Pete J., Bialkowski K., Tan A., Bierhaus A., Cooper M.E., Forbes J.M. (2008). Cardiac inflammation associated with a Western diet is mediated via activation of RAGE by AGEs. Am. J. Physiol. Endocrinol. Metab..

[17] Ilgenli T.F., Akpinar O. (2007). Acute effects of smoking on right ventricular function. Swiss Med. Wkly..

[18] van der Pol A., van Gilst W.H., Voors A.A., van der Meer P. (2019). Treating oxidative stress in heart failure: Past, present and future. Eur. J. Heart Fail..

[19] Singh R.B., Fedacko J., Goyal R., Rai R.H., Nandave M., Tonk R.K., Gaur S.S., Gautam R., Chibisov S. (2020). Pathophysiology and significance of troponin t, in heart failure, with reference to behavioural risk factors. World Heart J..

[20] Henein M.Y., Vancheri S., Longo G., Vancheri F. (2022). The Impact of Mental Stress on cardiovascular Health—Part II. J. Clin. Med..

[21] Suzuki H., Matsumoto Y., Kaneta T., Sugimura K., Takahashi J., Fukumoto Y., Takahashi S., Shimokawa H. (2014). Evidence for brain. activation in patients with Takotsubo cardiomyopathy. Circ. J..

[22] Pimple P., Lima B.B., Hammadah M. (2019). Psychological distress and subsequent cardiovascular events in individuals with coronary artery disease. J. Am. Heart Assoc..

[23] Song H., Fang F., Arnberg F.K., Mataix-Cols D., de la Cruz L.F., Almqvist C., Fall K., Lichtenstein P., Thorgeirsson G., Valdimarsdóttir U.A. (2019). Stress related disorders and risk of cardiovascular disease: Population based, sibling controlled cohort study. BMJ.

[24] Barbiero S., Aimo A., Castiglione V., Giannoni A., Vergaro G., Passino C., Emdin M. (2018). Healthy hearts at hectic pace: From daily life stress to abnormal cardiomyocyte function and arrhythmias. Eur. J. Prev. Cardiol..

[25] Aweimer A., Engemann L., Amar S., Ewers A., Afshari F., Maiß C., Kern K., Lücke T., Mügge A., El-Battrawy I. (2023). Stress-Mediated Abnormalities in Regional Myocardial Wall Motion in Young Women with a History of Psychological Trauma. J. Clin. Med..

[26] Cristine J.R., Tami A.M. (2015). Disruption of circadian rhythms and sleep on critical illness and the impact on cardiovascular events. Curr. Pharm. Des..

[27] Khayat R., Abraham W., Patt B. (2012). Central sleep apnea is a predictor of cardiac readmission in hospitalised patients with systolic heart failure. J. Card. Fail..

[28] Seyedian S.M., Majididi S., Asgharinejad L., Esmaeili M., Ahmadi F., Salemzadeh M. (2015). Acute and chronic effect of cigarette on right and left ventricular diastolic function. Jentashapir J. Health Res..

[29] Morimoto A., Tatsumi Y., Deura K., Mizuno S., Ohno Y., Watanabe S. (2013). Impact of cigarette smoking on impaired insulin secretion and insulin resistance in Japanese men: The Saku Study. J. Diabetes Investig..

[30] Zhang Y.B., Meng Y.H., Chang S., Zhang R.Y., Shi C. (2016). High fructose causes cardiac hypertrophy via mitochondrial signalling pathway. Am. J. Transl. Res..

[31] Santos C.X., Anilkumar N., Zhang M., Brewer A.C., Shah A.M. (2011). Redox signalling in cardiac myocytes. Free Radic. Biol. Med..

[32] Butler T.J., Ashford D., Seymour A.M. (2017). Western diet increases cardiac ceramide content in healthy and hypertrophied hearts. Nutr. Metab. Cardiovas. Dis..

[33] Singh R.B., Hristova K., Fedacko J., Singhal S., Khan S., Wilson D.W., Takahashi T., Sharma Z. (2015). Antioxidant vitamins and oxidative stress in chronic heart failure. World Heart J..

[34] Weir R.A.P., Tsorlalis I.K., Steedman T., Dargie H.J., Fraser R., McMurray J.J.V., Connell J.M.C. (2011). Aldosterone and cortisol predict medium-term left ventricular remodelling following myocardial infarction. Eur. J. Heart Fail..

[35] Singh R.B., Elkilany G., Visen A.S., Visen S.S. (2021). The puzzle of diastole and imbalance of risk factors and protective factors. World Heart J..

[36] Snelder S.M., de Groot-de Laat L.E., Biter L.U., Castro Cabezas M., Pouw N., Birnie E., Boxma-de Klerk B.M., Klaassen R.A., Zijlstra F., van Dalen B.M. (2020). Subclinical cardiac dysfunction in obesity patients is linked to autonomic dysfunction: Findings from the CARDIOBESE study. ESC Heart Fail..

[37] Huang J., Li G.A., Wang J., Jiao Y.W., Qian Z.F., Fan L., Tang L.M. (2023). Evaluation of subclinical left ventricular systolic dysfunction in obese patients by global myocardial work. Diabetol. Metab. Syndr..

[38] Elkilany G.E., Ghobashi A.S., Salama M., Singh J., Allah S.B., Elmahal M., Singh R., Nanda N.C. (2020). Prevalence and sub clinical detection of concomitant dilated cardiomyopathy in subjects with bicuspid aortic valves. J. Cardiol. Cardiovasc. Ther..

[39] Elkilany G.N., Baath Allah S., Lohana P., Sozzi F., Singh J., Khorshid M., Singh R.B., Aiash H. (2020). Sub-clinical detection of left ventricular myocardial dysfunction in valvular heart diseases: A state-of-the-art review in a speckle tracking echocardiography and myocardial performance. J. Cardiol. Res. Rev. Rep..

[40] Oh J.K., Park J.H. (2022). Role of strain echocardiography in patients with hypertension. Clin. Hypertens..

[41] Imbalzano E., Zito C., Carerj S., Oreto G., Mandraffino G., Cusmà-Piccione M., di Bella G., Saitta C., Saitta A. (2011). Left ventricular function in hypertension: New insight by speckle tracking echocardiography. Echocardiography.

[42] Kang S.J., Lim H.S., Choi B.J., Choi S.Y., Hwang G.S., Yoon M.H., Tahk S.J., Shin J.H. (2008). Longitudinal strain and torsion assessed by two-dimensional speckle tracking correlate with the serum level of tissue inhibitor of matrix metalloproteinase-1, a marker of myocardial fibrosis, in patients with hypertension. J. Am. Soc. Echocardiogr..

[43] Ghoreyshi-Hefzabad S.M., Jeyaprakash P., Vo H.Q., Gupta A., Ozawa K., Pathan F., Negishi K. (2023). Subclinical systolic dysfunction detected by 2D speckle tracking echocardiography in adults with diabetes mellitus: Systematic review and meta-analysis of 6668 individuals with diabetes mellitus and 7218 controls. Int. J. Cardiovasc. Imaging.

[44] Apostu A., Malita D., Arnautu S.F., Tomescu M.C., Gaiță D., Popescu A., Mare R., Gidea R., Arnautu D.A. (2023). Significant Association between Subclinical Left Cardiac Dysfunction and Liver Stiffness in Metabolic Syndrome Patients with Diabetes Mellitus and Non-Alcoholic Fatty Liver Disease. Medicina (Kaunas).

[45] Tsvetkov V.A., Krutikov E.S., Chistyakova S.I. (2020). Subclinical left ventricular dysfunction in patients with type 2 diabetes mellitus. Probl. Endokrinol..

[46] Simmonds S.J., Cuijpers I., Heymans S., Jones E.A.V. (2020). Cellular and molecular differences between HFpEF and HFrEF: A step ahead in an improved pathological understanding. Cells.

[47] Shin S.H., Suh Y.J., Baek Y.S., Lee M.J., Park S.D., Kwon S.W., Woo S.I., Kim D.H., Park K.S., Kwan J. (2016). Impact of area strain by 3D speckle tracking on clinical outcome in patients after acute myocardial infarction. Echocardiography.

[48] Sengupta P.P., Tajik A.J., Chandrasekaran K., Khandheria B.K. (2008). Twist mechanics of the left ventricle: Principles and application. JACC Cardiovasc. Imaging.

[49] Haugaa K.H., Dejgaard L.A. (2018). Global longitudinal strain: Ready for clinical use and guideline implementation. J. Am. Coll. Cardiol..

[50] Santoro C., Arpino G., Esposito R., Lembo M., Paciolla I., Cardalesi C., de Simone G., Trimarco B., De Placido S., Galderisi M. (2017). 2D and 3D strain for detection of subclinical anthracycline cardiotoxicity in breast cancer patients: A balance with feasibility. Eur. Heart J. Cardiovasc. Imaging.

[51] Takimoto E., Kass D.A. (2007). Role of oxidative stress in cardiac hypertrophy and remodeling. Hypertension.

[52] Singh R.B., Elkilany G., Fedacko J., Hristova K., Palmiero P., Singh J., Manal M.A., Badran H.M., Singh R.B., Fedacko J., Elkilany G., Hristova K. (2024). Evolution of the natural history of myocardial twist and diastolic dysfunction as cardiac dysfunction. Chronic Heart Failure, Pathophysiology and Management.

[53] Marwick T.H. (2013). Methods used for the assessment of LV systolic function: Common currency or tower of Babel?. Heart.

[54] Baath Allah S., Elmahal M., Askar M.H., Singh J., Khorshid M.H., Lohana P., Fedacko J., Elkilany G.E.N. (2020). Myocardial deformation imaging meta-analysis in two cohorts of patients from UAE and Heart Hospital Hamad Medical Corporation: A potential role in assessment of coronary artery disease severity and myocardial viability. J. Clin. Exp. Cardiolog..

[55] Johnson C., Kuyt K., Oxborough D., Stout M. (2019). Practical tips and tricks in measuring strain, strain rate and twist for the left and right ventricles. Echo. Res. Pract..

[56] Abou R., van der Bijl P., Bax J.J., Delgado V. (2020). Global longitudinal strain: Clinical use and prognostic implications in contemporary practice. Heart.

[57] Singh R.B., Torshin V.I., Chibisov S., Goyal R.K., Watanabe S., Nakagami H., Mogi M., Nayak B.N. (2019). Can protective factors inculcate molecular adaptations of cardiomyocyte in the prevention of chronic heart failure?. World Heart J..

[58] ICC, ICN, ISCN Committee (2023). 2023 ICC/ICN/ISC Guideline for the Management of Heart Failure: A Report of the International College of Cardiology/International College of Nutrition and Indian Society of Chrono Medicine, Joint Committee on Guidelines, for Developing and Newly Industrialized Countries. ICC, ICN, ISCN Committee. World Heart J..

[59] Aggrawal M., Bozkurt B., Panjrath G., Aggrawal B., Ostfield R.J., Barnard N.D., Gaggin H., Freemen A., Allen K., Madan S. (2018). Lifestyle modifications for preventing and treating heart failure. J. Amer. Coll. Cardiol..

[60] Singh R.B., Elkilany G., Hristova K., Fedacko J., Elmarghi O., Chakravorty S., Shukla A.K., Jain M., Agarwal A., Yaduvanshi A. (2022). Current controversies persisting in the recent guidelines for identification and classification of chronic heart failure. World Heart J..

[61] Khouri M.G., Peshock R.M., Ayers C.R., de Lemos J.A., Drazner M.H. (2010). A 4-tiered classification of left ventricular hypertrophy based on left ventricular geometry: The Dallas heart study. Circ. Cardiovasc. Imaging.

[62] Yuda S. (2021). Current clinical applications of speckle tracking echocardiography for assessment of left atrial function. J. Echocardiogr..

[63] Lacalzada J., de la Rosa A., Izquierdo M.M., Jiménez J.J., Iribarren J.L., García-González M.J., López B.M., Duque M.A., Barragán A., Hernández C. (2015). Left ventricular global longitudinal systolic strain predicts adverse remodeling and subsequent cardiac events in patients with acute myocardial infarction treated with primary percutaneous coronary intervention. Int. J. Cardiovasc. Imaging.

[64] Cong T., Sun Y., Shang Z., Wang K., Su D., Zhong L., Zhang S., Yang Y. (2015). Prognostic value of speckle tracking echocardiography in patients with ST-elevation myocardial infarction treated with late percutaneous intervention. Echocardiography.

[65] Hordern M.D., Coombes J.S., Cooney L.M., Jeffrises L., Prins J.B., Marwick T.H. (2009). Effects of exercise intervention on myocardial function in type 2 diabetes. Heart.

[66] Laffond A., Picon C.R., Rodriguez- Munoz P.M., Vela R.J., Vinhaspre-Hernandez R.R., Navas-Echavarria N., Sánchez-González J.L. (2023). Mediterranean diet for primary and secondary prevention of cardiovascular disease and mortality: An updated systematic review. Nutrients.

[67] American Heart Association American Heart Association Recommendations for Physical Activity in Adults and Kids. https://www.heart.org/en/healthy-living/fitness/fitness-basics/aha-recs-for-physical-activity-in-adults.

